# General Practitioners' Experiences With Implementing Colon or Prostate Cancer Survivorship Care in Primary Care: A Cross-Sectional Survey Nested Within Two Randomized-Controlled Trials

**DOI:** 10.1200/OP-24-00491

**Published:** 2024-12-02

**Authors:** Julien A.M. Vos, Barbara M. Wollersheim, Henk C.P.M. van Weert, Lonneke V. van de Poll-Franse, Kristel M. van Asselt

**Affiliations:** ^1^ Amsterdam UMC, University of Amsterdam, Department of General Practice, Amsterdam, the Netherlands; ^2^ Amsterdam Public Health, Research Programme Quality of Care, and Personalized Medicine, Amsterdam, the Netherlands; ^3^ Division of Psychosocial Research and Epidemiology, The Netherlands Cancer Institute, Antoni van Leeuwenhoek Hospital, Amsterdam, the Netherlands; ^4^ Department of Research, Netherlands Comprehensive Cancer Organization (IKNL), Utrecht, the Netherlands; ^5^ Department of Medical and Clinical Psychology, CoRPS – Center of Research on Psychology in Somatic Diseases, Tilburg University, Tilburg, the Netherlands; ^6^ Department of General Practice and Nursing Science, Julius Center for Health Sciences and Primary Care, University Medical Center Utrecht, Utrecht, the Netherlands

## Abstract

**PURPOSE:**

To evaluate general practitioners' (GPs) experiences with providing cancer survivorship care and explore readiness for implementation.

**METHODS:**

This cross-sectional survey study was nested within two randomized-controlled trials conducted in the Netherlands between 2015 and 2023, comparing GP- with specialist-led survivorship care for patients with colon or prostate cancer. An adapted version of the normalisation measure development (NoMAD) survey was distributed among participating GPs. NoMAD assesses the implementation of complex interventions and includes seven items on experiences (score ranges, 0-10) and 19 core items (expressed as % agreement). Higher scores indicate greater normalization, that is, embedding in primary care.

**RESULTS:**

In total, 214 GPs participated (response rate, 69%). The overall experience with providing survivorship care was 7.0 ± 1.6 for prostate cancer and 6.4 ± 1.8 for colon cancer. Lowest scores were seen for willingness to provide care (5.9 ± 2.4 and 5.0 ± 2.5, respectively), expected future involvement (6.6 ± 2.0 and 5.6 ± 2.5), and appropriateness of involvement (6.4 ± 2.1 and 5.6 ± 2.7). GPs in both trials agreed (±75%) there was potential value for patients, but not for their own work (±50%). Survivorship care for colon cancer was often perceived as different from usual care (74%). GPs' self-reported knowledge of care was high in the prostate cancer trial (62%), but not in the colon cancer trial (41%). GPs from both trials agreed that they could easily integrate management of physical and psychosocial effects into their work (±70%), but integrating routine check-ups was rated less positively (±55%). Financial compensation was deemed necessary (±80% agreed). Twenty-one percent was willing to provide care for other cancer types.

**CONCLUSION:**

GPs recognize value of providing survivorship care for patients, but experiences differ on the basis of cancer type. Implementation of prostate versus colon cancer survivorship care appears more feasible.

## BACKGROUND

Worldwide, the number of cancer survivors is increasing.^1^ Cancer survivors experience a wide variety of chronic health conditions and symptoms for which they require care.^2^ Health care providers aim to help patients manage the (long-term and late) effects of cancers and its treatment. Cancer survivorship care focuses on the health and well-being of persons with cancer from the time of diagnosis.^3^ It involves several components, including the prevention and surveillance for recurrences and new cancers, surveillance and management of physical and psychosocial effects, surveillance and management of chronic medical conditions, health promotion and disease prevention, and care co-ordination and communication.^4^

**CONTEXT**

**Key Objective**
To evaluate general practitioners' (GPs) experiences with providing colon or prostate cancer survivorship care and to assess their readiness for implementing this care in primary settings.
**Knowledge Generated**
GPs recognized the value of providing survivorship care for patients with cancer, although their experiences varied by cancer type. Overall, GPs reported more positive experiences with prostate cancer survivorship care.
**Relevance**
From the GPs' perspective, the implementation of prostate cancer survivorship care appears more feasible. To improve the success of implementation in primary care, several factors are necessary, including tailored education, automatic monitoring systems, and appropriate financial compensation.

Cancer survivorship practices differ greatly around the globe.^5^ In most Western countries, like the Netherlands, survivorship care is provided by specialists in the hospitals. Although specialist-led survivorship care is most common, its effectiveness and sustainability to comprehensively treat cancer survivors have been questioned.^6^ Cancer survivors frequently experience unmet needs and visit other health care providers than their treating specialists,^7,8^ resulting in the fragmentation of care. These issues have led to the investigation of other models of survivorship care.^9^ Because patients frequently visit their general practitioner (GP) for cancer-related and for other problems due to comorbidities, it has been suggested that the GP could take on a greater role in cancer survivorship care.^10,11^ GP- versus specialist-led survivorship care appears to be at least as effective in terms of clinical- and patient-reported outcomes while generally resulting in lower health care costs.^12^ However, implementing survivorship care in primary care also faces numerous barriers, including a lack of resources and time for GPs to provide comprehensive care, and limited GP knowledge about survivorship care practices.^13,14^ Potential solutions to combat these issues, such as providing survivorship resources and bolstering GP education, have been largely based on GPs' perspectives, rather than actual experiences with providing care.

Over the past 2 decades, only a few randomized-controlled trials have compared GP- with specialist-led survivorship care.^12^ Two of these trials were conducted in the Netherlands, which focused on patients with colon cancer (2015-2023)^15^ and prostate cancer (2018-2022).^16^ These two trials offered a valuable opportunity to assess GPs' initial experiences with implementing cancer survivorship care. This survey study was conducted among the participating GPs of both trials, aiming to evaluate their experiences and to explore their readiness for implementing this type of care in primary settings. These insights may help guide a potential care transition from the hospital to primary care.

## METHODS

### Study Design

This cross-sectional survey study was nested within two randomized-controlled trials, namely, the I CARE and the PROSPEC study.^15,16^ I CARE focused on patients with colon cancer and involved a follow-up duration of 5 years,^15^ whereas the PROSPEC study focused on patients with prostate cancer and involved a follow-up duration of 2 years.^16^ In both trials, patients were randomly assigned after cancer treatment (generally at the first follow-up visit) to receive survivorship care from either their GP or treating specialist. In the I CARE study, 43 (15%) of 293 GPs declined participation.^17^ In the PROSPEC study, 22 (6%) of 398 GPs declined. The primary outcomes of these studies have been published in previous papers (Wollersheim et al, manuscript submitted for publication).^18^ The study protocols were approved by the medical ethics committee of the Academic Medical Center (Amsterdam, the Netherlands; MEC 2014_332) and the Institutional Review Board of the Antoni van Leeuwenhoek Hospital (Amsterdam, the Netherlands; METC18.0033/M17PRO).

### Data Collection

GPs assigned to provide survivorship care were invited through e-mail or post to fill out the survey at the end of trial participation. Invitations were sent after the follow-up of their patient had finished. Completion of the survey was voluntary. GPs who had provided survivorship care for more than one patient only received the invitation once. In some cases, the patient had already transferred back to the specialist or to a new GP during follow-up. GPs who had never provided survivorship care were therefore excluded from participation. GPs who did not respond to the invitation received at least one reminder, but no other follow-up strategies were used. Data were recorded using Castor electronic data capture.^19^

### Outcomes

A validated Dutch translation of the normalisation measure development (NoMAD) survey was distributed among GPs.^20^ NoMAD is based on normalisation process theory (NPT) and measures the four core constructs of NPT, that is, coherence (CO), cognitive participation (CP), collective action (CA), and reflexive monitoring (RM). CO refers to the question whether GPs made sense of survivorship care (n = 4 items), CP addresses the GPs' engagement with survivorship care (n = 6), CA assesses the work that GPs did to enable survivorship care (n = 5), and RM is the GPs' appraisal of the effect of providing survivorship care (n = 4). Higher scores indicate greater normalization, that is, the embedding of cancer survivorship care in primary care. In line with the developers' recommendations, the NoMAD survey was adapted to fit the purpose of the intervention.^21,22^ Parallel to the I CARE study, semi-structured interviews were held with 17 GPs to explore their experiences with providing colon cancer survivorship care.^23^ On the basis of the results of this qualitative study, the NoMAD was customized and subsequently used in exactly the same way in the PROSPEC study. The survey consists of three parts, summarized below. The complete survey is available in the Data Supplement (online only).

1. Part A consists of demographic questions, such as the age of GPs and their years in clinical practice;
2. Part B measures GPs' experiences with providing care using scale items (scores ranging from 0 to 10). In this section, we added general questions on the content of follow-up consultations and included four additional scale items, such as willingness to provide survivorship care and overall experience with care;
3. Part C includes 19 statements related to the NPT core constructs, where GPs rate their agreement on a five-point Likert scale (ranging from [0] totally disagree to [5] totally agree). In this section, we rephrased core items to fit the purpose of the intervention and removed items related to teamwork or collaboration (as no other health care professionals were involved). We also included questions on future recommendations, such as the need for additional education and the need for financial compensation;
4. Finally, we included an open-text field item at the end of the survey in which GPs could elaborate and provide extra information.

### Statistical Analysis

Descriptive statistics were used to describe baseline characteristics and survey items. Data from the I CARE and PROSPEC study were analyzed separately to explore potential differences in colon and prostate cancer survivorship care. To facilitate presentation and interpretation, disagree and strongly disagree were merged into disagree, and agree and strongly disagree into agree. For each NPT core construct, we calculated Cronbach's alpha (α). Internal consistency was acceptable for CO (α = .71), CP (α = .78), and CA (α = .76), but questionable for RM (α = .60). The final open-text field item was described thematically. Themes were derived from the item by one researcher (J.A.M.V.). All data were analyzed using IBM SPSS Statistics for Windows, version 25, Armonk, NY. *P* < .05 were considered statistically significant. The written informed consent was obtained from all participants involved in this study.

## RESULTS

**FIG 1.**
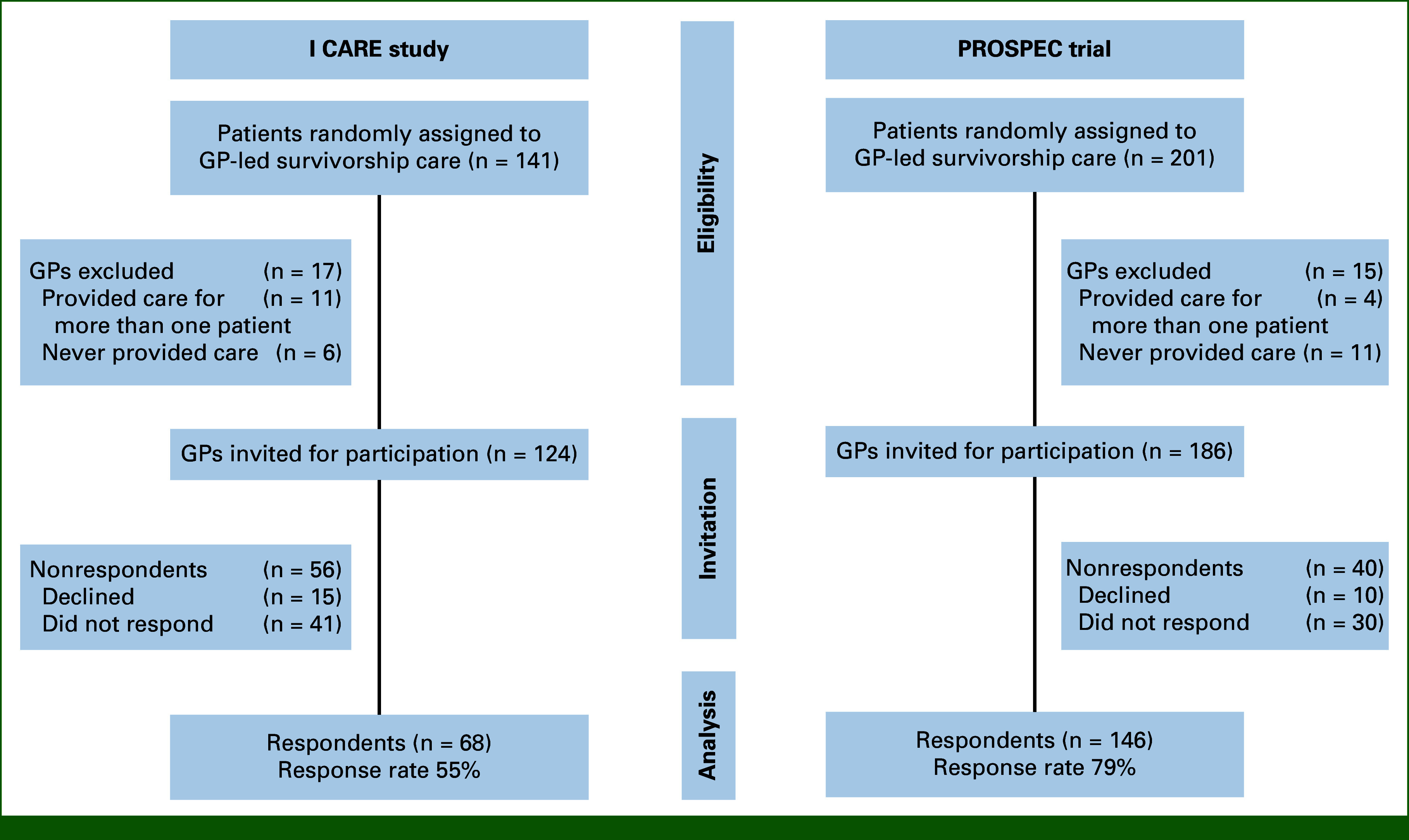
Flow diagram of participation in the cross-sectional study. Participation was declined due to various reasons (GP was absent or does not work in the practice anymore [n = 20], too time-consuming [n = 3], GP was only briefly involved [n = 1]). GP, general practitioner.

In total, 214 GPs filled out the survey (Fig 1 shows the flow diagram of participation). In the I CARE study, the response rate was 55% (68 of 124 GPs) and in the PROSPEC study 79% (146 of 186). GPs had a mean age of 52.1 years (±9.6) and, on average, had spent 19.0 years (±9.5) in general practice (Table 1).

**TABLE 1.** Baseline Characteristics

Characteristic	Overall N = 214	I CARE Study (colon cancer) n = 68	PROSPEC Study (prostate cancer) n = 146
Age in years, mean (SD)	52.1 (9.6)	51.3 (9.2)	52.5 (9.8)
No. of years in general practice, mean (SD)	19.0 (9.5)	19.4 (8.6)	18.8 (9.9)
Size of the practice population, median (IQR)	3,000 (1,800)	3,000 (1,800)	3,025 (1,700)
No. of GPs working in the practice, mean (SD)	3.6 (2.1)	3.7 (2.1)	3.6 (2.1)

Abbreviations: GP, general practitioner; SD, standard deviation.

### Implementing Colon Cancer Survivorship Care

GPs rated their overall experience at 6.4 ± 1.8 on a scale to 10 (Table 2). The highest ratings were for managing psychosocial effects (7.3 ± 1.6) and physical effects (6.6 ± 2.0), whereas the lowest was for willingness to provide survivorship care to all patients with colon cancer (5.0 ± 2.8). During follow-up consultations, GPs most frequently discussed follow-up test results (85%) and physical effects of cancer and its treatment (76%). Lifestyle and prevention was discussed least often (42%).

**TABLE 2.** Outcomes of the Normalisation Measure Development Survey

Outcome	I CARE Study (colon cancer) n = 68	PROSPEC Study (prostate cancer) n = 146
Survey part B		
Experiences with survivorship care, mean (SD)		
Overall experience	6.4 (1.8)	7.0 (1.6)
Confidence in organizing follow-up	6.3 (2.2)	7.1 (1.7)
Confidence in managing physical effects	6.6 (2.0)	7.3 (1.4)
Confidence in managing psychosocial effects	7.3 (1.6)	7.5 (1.3)
Appropriateness of GP involvement	5.6 (2.7)	6.4 (2.1)
Willingness to provide survivorship care to all patients with colon/prostate cancer	5.0 (2.8)	5.9 (2.4)
Expected future GP involvement	5.6 (2.5)	6.6 (2.0)
Content of follow-up consultations, yes, No. (%)		
Follow-up test results	57 (85)	145 (99)
Physical effects	51 (76)	132 (90)
Psychosocial effects	42 (63)	106 (73)
Lifestyle and prevention	28 (42)	78 (54)
Other, No. (%)	2 (3)	16 (11)
Survey part C		
NPT construct scores, median (IQR)		
CO	3.3 (1.0)	3.7 (1.0)
CP	3.3 (0.8)	3.7 (0.8)
CA	3.8 (0.8)	3.8 (0.6)
RM	3.3 (1.0)	3.7 (0.7)
Recommendations for implementation, No. (%)		
Willing to provide survivorship care for other cancer types	12 (21)	29 (22)
Need for additional education	27 (49)	63 (47)
Need for a monitoring system	44 (79)	90 (67)
Need for a multidisciplinary approach	31 (56)	66 (49)
Need for financial compensation	45 (82)	108 (80)

Abbreviations: CA, collective action; CO, coherence; CP, cognitive participation; GP, general practitioner; NPT, normalisation process theory; RM, reflexive monitoring; SD, standard deviation.

**FIG 2.**
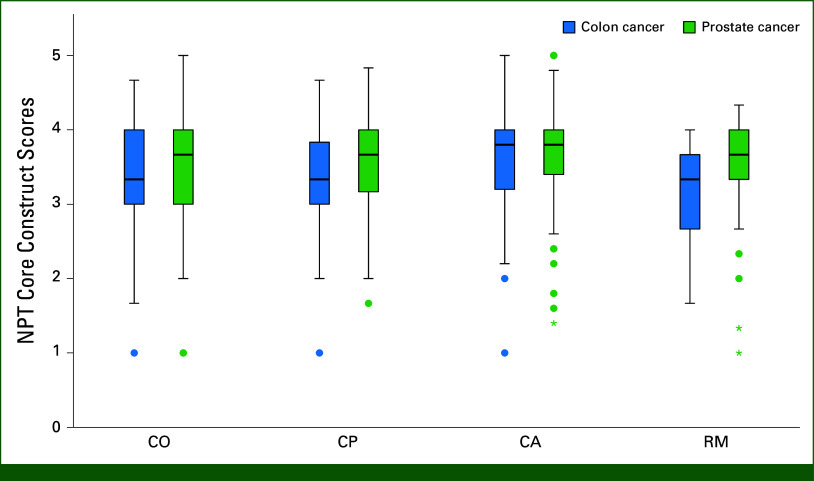
Box plot of NPT core construct scores. CA, collective action; CO, coherence; CP, cognitive participation; NPT, normalisation process theory; RM, reflexive monitoring.

**FIG 3.**
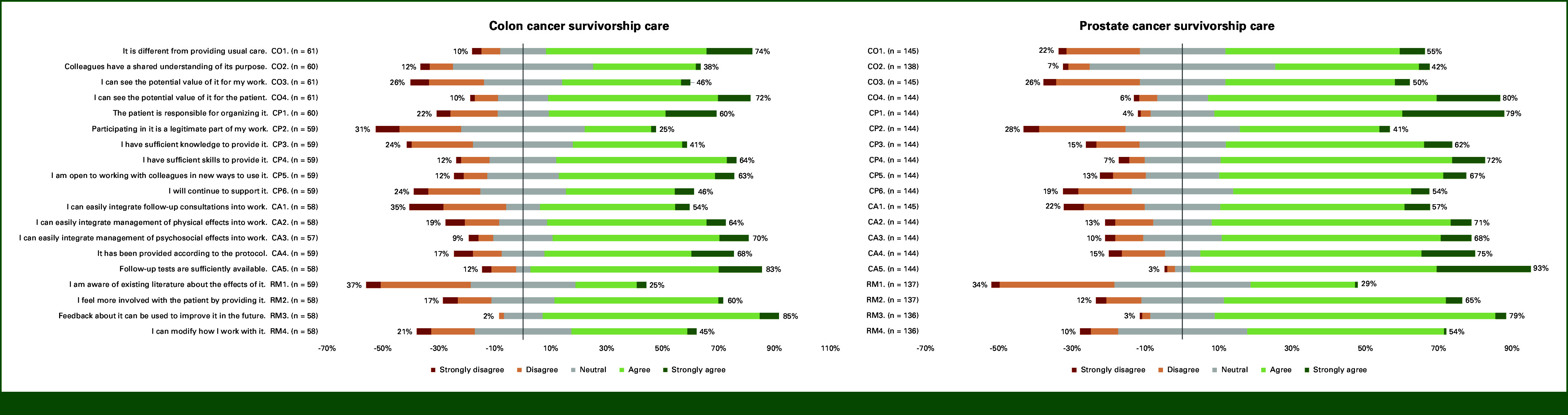
Frequency distribution of item responses. CA, collective action; CO, coherence; CP, cognitive participation; RM, reflexive monitoring.

Figure 2 presents a box plot of NPT core construct scores, with the highest agreement in the CA construct (median of 3.8 [IQR, 1.0]). Other core constructs had a median score of 3.3. Figure 3 shows the individual item responses. In the CO construct, 74% of GPs agreed that providing colon cancer survivorship care differs from usual care, with 72% recognizing potential value for patients, but only 46% seeing value for themselves. In the CP construct, 60% agreed that patients are responsible for organizing care. In all, 64% of GPs felt they had sufficient skills to provide colon cancer survivorship care, but 41% felt they had sufficient knowledge. Only 25% agreed that this care is a legitimate part of their work. In the CA construct, 64%-70% agreed they could integrate the management of physical and psychosocial effects into their work, but 54% felt they could integrate follow-up consultations (ie, routine check-ups). Finally, in the RM construct, 60% felt more involved with patients through providing survivorship care. Only 25% was aware of the existing literature.

### Implementing Prostate Cancer Survivorship Care

The overall experience was rated at 7.0 ± 1.6 on a scale to 10. The highest ratings were for managing psychosocial effects (7.5 ± 1.3), physical effects (7.3 ± 1.4), and organizing follow-up (7.1 ± 1.7). The lowest rating was for willingness to provide survivorship care (5.9 ± 2.4). Nearly all GPs discussed follow-up test results (99%) and physical effects (90%) during consultations, whereas lifestyle and prevention were less frequently addressed (54%). In the open-text field items, some GPs indicated that they also discussed the effects on sexual functioning.

GPs reported similar level agreement across all NPT core constructs (Fig 2). In CO, 55% agreed that providing prostate cancer survivorship care differs from usual care. Eighty percent recognized potential value for patients, but only 50% saw value for themselves (Fig 3). In CP, 79% agreed that patients are responsible for organizing care. In total, 62%-72% of GPs felt they had sufficient knowledge and skills to provide prostate cancer survivorship care, but only 41% agreed that this is a legitimate part of their work. In CA, GPs agreed with most items, although only 57% agreed they could easily integrate follow-up consultations into their work. In RM, 65% felt more involved with their patients. Only 29% was aware of the existing literature.

### Recommendations for Implementing Cancer Survivorship Care

Of all the GPs, 21% were willing to provide survivorship care for more types of cancer (Table 2). Approximately 50% of GPs were in need of additional education about survivorship care. GPs frequently indicated the need for an automatic monitoring system (67% in the prostate *v* 79% in the colon cancer trial), but not for a multidisciplinary approach (49% *v* 56%). GPs in both trials highlighted the need for financial compensation (±80% agreed). GPs were also asked to estimate the amount of money needed per patient per year to provide survivorship care. Only a few GPs answered this question, but the estimated amount was lower for prostate (n = 46; median, €100; IQR, 70) compared with colon cancer (n = 14; median, €175; IQR, 288).

The final open-ended question of the survey yielded a variety of comments (n = 95). Most GPs discussed barriers to provide survivorship care in primary care, including insufficient time and resources (n = 56 [59%]). One GP mentioned, “I think the GP is very capable of providing this type of care. However, if this becomes the standard for multiple diseases, then we do not have the capacity for it. That is a prerequisite.” (GP, 40 years, prostate cancer trial). Some GPs expressed doubts about their competences and highlighted the need for education: “this was a patient who was stable. The problem arises if there are any [cancer-related] issues. That's where I don't feel competent.” (GP, 57 years, prostate cancer trial). Some GPs also described the important proactive role of the patient in organizing follow-up care (n = 18 [19%]).

## DISCUSSION

In this cross-sectional survey study, GPs' experiences with providing cancer survivorship care in primary care were described. Differences were seen in providing prostate versus colon cancer survivorship care. This finding underscores the importance of recognizing and differentiating cancer types in survivorship care. On the basis of the survey results, implementation of prostate cancer survivorship care seems more feasible. Several requirements were described to improve the success rate of implementation in primary care, such as the need for tailored education, an automatic monitoring system, and financial compensation.

To our knowledge, this is the first study that used the NoMAD survey to explore implementation potential of cancer survivorship care in primary care. In the absence of reference studies, we can only compare the results of the two trials against one another. Interestingly, GPs in the prostate versus colon cancer trial reported better (or similar) scores on nearly all survey items. There are a few explanations for this finding. First, the overall participation rate was much higher in the PROSPEC versus I CARE study (69% *v* 25%; Wollersheim et al, manuscript submitted for publication).^17^ In the I CARE study, reasons to decline participation were most often related to the research question (57%), which may reflect the lower support base for implementing colon cancer survivorship care and explain the poorer survey results.^17^ Next, there is an important difference in the complexity and intensity of the follow-up schedule after prostate versus colon cancer. During the study period, patients with prostate cancer only required prostate-specific antigen (PSA) monitoring,^24^ whereas patients with colon cancer typically required multiple blood tests (carcinoembryonic antigen [CEA] monitoring), combined with repeated abdominal ultrasounds and colonoscopies.^25^ It is therefore not surprising that implementing prostate cancer survivorship care could be considered easier. In 2017, the colon cancer follow-up guideline was revised (ie, ultrasounds are no longer needed), which would make it easier to implement colon cancer follow-up as well.^25^ Despite this revision of the guideline, GPs are still more familiar with providing PSA versus CEA monitoring, since PSA is also used for opportunistic prostate cancer screening.^26^ The GPs' familiarity with PSA monitoring corresponds with the fact that prostate cancer survivorship care was less often perceived as different from usual care (55% *v* 74%). Additionally, GPs' self-reported knowledge of survivorship care was higher in the prostate cancer group (62% *v* 41%). Limited knowledge is a well-known barrier to providing cancer survivorship care in primary care.^13^ To address these knowledge gaps, it is important to provide tailored education and training, particularly in colon survivorship care, if such care were to be implemented in primary care.^27^

On the basis of a previous qualitative study, the NoMAD survey was adapted to fit the purpose of the intervention and directly target the respondents, providing practical insights, including recommendations for implementation.^23^ The majority of GPs (81%) agreed on the need for financial compensation (81%), which is not surprising given that providing cancer survivorship care requires extra time and resources, which are currently lacking in primary care settings. These barriers were often mentioned in the open-ended final question. Addressing this issue would require the Dutch Health Care Authority (or a similar organization in other countries) to establish new tariffs that cover all associated costs. The majority of GPs (73%) also supported the need for an automatic monitoring system. These systems can notify patients and GPs when it is time for a new appointment, enhancing the organization of care. Interestingly, a recent systematic review on barriers and solutions to implementation of GP-led survivorship care did not identify this need.^13^ However, such systems can significantly ease the implementation of survivorship care in primary care. For example, a previous trial has demonstrated that remote surveillance of prostate cancer (in which the patient does not have scheduled follow-up visits, but is facilitated through an online system) leads to comparable patient-reported outcomes in all respects.^28^ A similar trial is conducted for patients with colorectal cancer, with results expected soon.^29^ Future studies should explore the role of automatic monitoring systems in improving survivorship care.

Most previous studies have captured GPs' perceptions of providing cancer survivorship care, rather than their experiences.^11^ This is one of the first studies that investigated GPs' experiences with providing care. As the study included GPs from two different randomized-controlled trials, involving different types of cancer, it provides a unique perspective on survivorship care. However, several limitations must be considered. First, systematic differences between the two trials, such as the varying follow-up duration (2 years *v* 5 years), could significantly affect the survey outcomes. Consequently, comparisons between the two trials should be interpreted with caution. Nevertheless, there are numerous similarities between the two studies that make comparison valuable, including their overlapping time frames, shared pragmatic approach, and use of the identical NoMAD survey. Second, because this study was conducted among GPs who agreed to be randomly assigned for survivorship care, it may have led to an over-representation of GPs who were generally positive about the intervention. Despite that, the survey items showed conflicting responses, so the findings are likely to reflect both positive and negative experiences.

In conclusion, our survey study highlighted both the potential and challenges associated with the transition of cancer survivorship to primary care. It revealed valuable insights into the barriers to implementation, such as the need for financial compensation and tailored education. Addressing these barriers is important to ensuring the success of any potential transition. From the GPs' perspective, implementation of prostate versus colon cancer survivorship care seems more feasible. Recognizing the differences between the cancer types is crucial, indicating that a one-size-fits-all approach may not be appropriate.

## Data Availability

A data sharing statement provided by the authors is available with this article at DOI https://doi.org/10.1200/OP-24-00491.

## References

[1] Sung H, Ferlay J, Siegel RL, et al: Global cancer statistics 2020: GLOBOCAN estimates of incidence and mortality worldwide for 36 cancers in 185 countries. CA Cancer J Clin 71:209-249, 2021 33538338 10.3322/caac.21660

[2] Wu HS, Harden JK: Symptom burden and quality of life in survivorship: A review of the literature. Cancer Nurs 38:E29-E54, 2015 24831042 10.1097/NCC.0000000000000135

[3] Hewitt M, Greenfield S, Stovall E: From Cancer Patient to Cancer Survivor: Lost in Transition. Washington, DC, National Academies Press, 2006

[4] Nekhlyudov L, Mollica MA, Jacobsen PB, et al: Developing a quality of cancer survivorship care framework: Implications for clinical care, research, and policy. J Natl Cancer Inst 111:1120-1130, 2019 31095326 10.1093/jnci/djz089 PMC6855988

[5] Mollica MA, Mayer DK, Oeffinger KC, et al: Follow-up care for breast and colorectal cancer across the globe: Survey findings from 27 countries. JCO Glob Oncol 6:1394-1411, 2020 32955943 10.1200/GO.20.00180 PMC7529533

[6] Jefford M, Howell D, Li Q, et al: Improved models of care for cancer survivors. Lancet 399:1551-1560, 2022 35430022 10.1016/S0140-6736(22)00306-3 PMC9009839

[7] Harrison JD, Young JM, Price MA, et al: What are the unmet supportive care needs of people with cancer? A systematic review. Support Care Cancer 17:1117-1128, 2009 19319577 10.1007/s00520-009-0615-5

[8] Hart NH, Crawford-Williams F, Crichton M, et al: Unmet supportive care needs of people with advanced cancer and their caregivers: A systematic scoping review. Crit Rev Oncol Hematol 176:103728, 2022 35662585 10.1016/j.critrevonc.2022.103728

[9] Chan RJ, Crawford-Williams F, Crichton M, et al: Effectiveness and implementation of models of cancer survivorship care: An overview of systematic reviews. J Cancer Surviv 17:197-221, 2023 34786652 10.1007/s11764-021-01128-1 PMC8594645

[10] Emery JD, Shaw K, Williams B, et al: The role of primary care in early detection and follow-up of cancer. Nat Rev Clin Oncol 11:38-48, 2014 24247164 10.1038/nrclinonc.2013.212

[11] Meiklejohn JA, Mimery A, Martin JH, et al: The role of the GP in follow-up cancer care: A systematic literature review. J Cancer Surviv 10:990-1011, 2016 27138994 10.1007/s11764-016-0545-4

[12] Vos JAM, Wieldraaijer T, van Weert H, et al: Survivorship care for cancer patients in primary versus secondary care: A systematic review. J Cancer Surviv 15:66-76, 2021 32815087 10.1007/s11764-020-00911-w PMC7822798

[13] Hayes BD, Young HG, Atrchian S, et al: Primary care provider–led cancer survivorship care in the first 5 years following initial cancer treatment: A scoping review of the barriers and solutions to implementation. J Cancer Surviv 18:352-365, 2022 36376712 10.1007/s11764-022-01268-y

[14] Lawrence RA, McLoone JK, Wakefield CE, et al: Primary care physicians' perspectives of their role in cancer care: A systematic review. J Gen Intern Med 31:1222-1236, 2016 27220499 10.1007/s11606-016-3746-7 PMC5023605

[15] Duineveld LA, Wieldraaijer T, van Asselt KM, et al: Improving care after colon cancer treatment in the Netherlands, personalised care to enhance quality of life (I CARE study): Study protocol for a randomised controlled trial. Trials 16:284, 2015 26112050 10.1186/s13063-015-0798-7 PMC4499213

[16] Wollersheim BM, van Asselt KM, van der Poel HG, et al: Design of the PROstate cancer follow-up care in Secondary and Primary hEalth Care study (PROSPEC): A randomized controlled trial to evaluate the effectiveness of primary care-based follow-up of localized prostate cancer survivors. BMC Cancer 20:635, 2020 32641023 10.1186/s12885-020-07112-9 PMC7346492

[17] Duineveld LAM, Vos JAM, Wieldraaijer T, et al: Recruitment challenges to the I CARE study: A randomised trial on general practitioner-led colon cancer survivorship care. BMJ Open 11:e048985, 2021 10.1136/bmjopen-2021-048985 PMC8386209 34429313

[18] Vos JAM, Duineveld LAM, Wieldraaijer T, et al: Effect of general practitioner-led versus surgeon-led colon cancer survivorship care, with or without eHealth support, on quality of life (I CARE): An interim analysis of 1-year results of a randomised, controlled trial. Lancet Oncol 22:1175-1187, 2021 34224671 10.1016/S1470-2045(21)00273-4

[19] Castor EDC: Castor electronic data capture, 2019. https://www.castoredc.com

[20] Vis C, Ruwaard J, Finch T, et al: Toward an objective assessment of implementation processes for innovations in health care: Psychometric evaluation of the Normalization MeAsure Development (NoMAD) questionnaire among mental health care professionals. J Med Internet Res 21:e12376, 2019 30785402 10.2196/12376 PMC6401675

[21] Rapley T, Girling M, Mair FS, et al: Improving the normalization of complex interventions: Part 1 – development of the NoMAD instrument for assessing implementation work based on normalization process theory (NPT). BMC Med Res Methodol 18:133, 2018 30442093 10.1186/s12874-018-0590-y PMC6238361

[22] Finch TL, Girling M, May CR, et al: Improving the normalization of complex interventions: Part 2 – validation of the NoMAD instrument for assessing implementation work based on normalization process theory (NPT). BMC Med Res Methodol 18:135, 2018 30442094 10.1186/s12874-018-0591-x PMC6238372

[23] Vos JAM, de Best R, Duineveld LAM, et al: Delivering colon cancer survivorship care in primary care; a qualitative study on the experiences of general practitioners. BMC Prim Care 23:13, 2022 35172743 10.1186/s12875-021-01610-w PMC8761520

[24] National guideline prostate carcinoma. https://richtlijnendatabase.nl/richtlijn/prostaatcarcinoom/follow_up.html

[25] National guideline colorectal carcinoma (CRC). https://richtlijnendatabase.nl/richtlijn/colorectaal_carcinoom_crc/startpagina_-_crc.html

[26] Kappen S, Koops L, Jürgens V, et al: General practitioners' approaches to prostate-specific antigen testing in the north-east of the Netherlands. BMC Fam Pract 21:270, 2020 33334312 10.1186/s12875-020-01350-3 PMC7747401

[27] Vos JAM, Wollersheim BM, Cooke A, et al: Primary care physicians’ knowledge and confidence in providing cancer survivorship care: A systematic review. J Cancer Surviv 18:1557-1573, 2023 37171716 10.1007/s11764-023-01397-y PMC11424677

[28] Frankland J, Brodie H, Cooke D, et al: Follow-up care after treatment for prostate cancer: Evaluation of a supported self-management and remote surveillance programme. BMC Cancer 19:368, 2019 31014282 10.1186/s12885-019-5561-0 PMC6480799

[29] Netherlands Trial Register: DISTANCE trial, 2023. https://trialsearch.who.int/Trial2.aspx?TrialID=NL9266

